# Global susceptibility and response to noncommunicable diseases

**DOI:** 10.2471/BLT.17.206763

**Published:** 2018-06-12

**Authors:** Arian Hatefi, Luke N Allen, Thomas J Bollyky, Sarah A Roache, Rachel Nugent

**Affiliations:** a Institute for Global Health Sciences and the Division of Hospital Medicine, University of California San Francisco, Box 0131, 533 Parnassus Ave, Room U126, San Francisco, CA 941143-0131, United States of America (USA).; b Nuffield Department of Primary Health Care Sciences, University of Oxford, Oxford, England.; c The Council on Foreign Relations, Washington, DC, USA.; d O’Neill Institute for National and Global Health Law, Georgetown University, Washington, DC, USA.; e Research Triangle Institute (RTI) International, Durham, USA.

Globalization and human interdependence have created immeasurable value for humanity. These forces, however, also provide channels for health risks to spread throughout the world. Global functions for health, such as international partnerships or research and development, are a rational response to global health risks like pandemics or globalized supply chains. Self-interest compels governments, or donors, to provide global functions even though their benefits are widely shared the world over.

Policy-makers and donors more clearly perceive win-win strategies for investment in global functions for infectious diseases, than for noncommunicable diseases. However, noncommunicable diseases account for two-thirds of low- and middle-income country deaths, but receive only 2% of donor assistance, while 36% is allocated to human immunodeficiency virus (HIV), tuberculosis and malaria.^1^^,^^2^ One explanation for this donor imbalance is that security from infectious diseases is viewed as a global public good that requires global cooperation and foreign assistance, whereas security from noncommunicable diseases is seen as a costly private good, best relegated to domestic health response. That is, global interdependence is widely accepted for infectious diseases, but not for noncommunicable diseases.

Reframing noncommunicable diseases as shared health threats with global interdependence, would justify global function provision for such diseases as a core donor response.

## Global susceptibility and risk

Societal structures that exploit human vulnerabilities, rather than individual choices or chance, limit our ability to avoid the major noncommunicable disease risks. Among these risks are four groups of factors: social determinants; behavioural biology; commercial determinants; and the physical environment.

First, noncommunicable disease susceptibility does not only arise from bad choices and chance. While social determinants describe a poverty-health nexus, noncommunicable diseases affect all socioeconomic groups, albeit in different ways.^3^ Social contagion and social disadvantage, rather than individual choices, are more to blame for noncommunicable disease susceptibility. Indeed, both risk factors and noncommunicable diseases spread through social networks.^4^ Social networks, built through person to person interactions or at larger scales, through media and digital social networks, are the backbone of human culture, but the influence they exert can also provide channels for health risks to exploit our vulnerabilities.

Second, behaviours of stress and self-preservation leave us vulnerable to social influence and are powerful drivers of many modifiable noncommunicable disease risks, for example tobacco or food consumption patterns. Social and neurocognitive susceptibility decrease, rather than determine, our ability to avoid high-risk exposures, especially in the most vulnerable groups, such as the young or disadvantaged.^5^

Third, increasingly global phenomena exploit our vulnerabilities. Corporations project global influence through marketing, supply chains, lobbying and social responsibility campaigns. These actions can be productive when aligned with public health interests, such as optimizing essential medicine use, but they can harm those susceptible when they are not, for instance tobacco use and excessive alcohol and processed food consumption, and fossil fuel use.^6^

Finally, the physical environments in which humans live and work, strongly influence noncommunicable disease prevalence.^7^ Sedentarism and calorie-rich diets are signs of a shared, global drift towards unhealthier lifestyles across countries.

How an interdependent international community shares the consequences of the rising noncommunicable disease burden is not completely understood. Noncommunicable diseases exact an immense drag on national economies. Projected cumulative losses are of 47 trillion United States dollars through 2030,^8^ but it is unclear how these costs will shape global and regional political, social and economic landscapes.

## The rationale for global functions

**Fig. 1:**
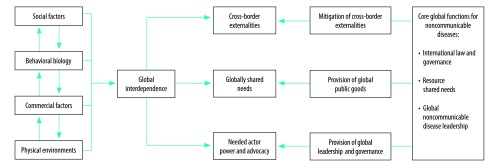
Rationalizing the case for global functions for noncommunicable diseases

The increase of noncommunicable diseases is a consequence of global susceptibility and multiplying risk factor exposure. All countries are at risk, because globalization is practically impossible to avoid. Relying on national responses and appealing to personal responsibility to control a global problem is insufficient. An effective noncommunicable disease response requires a full range of tools including global functions, which are cooperative strategies that transcend national sovereignty to solve global problems (Fig. 1).

Global functions can target three aspects of noncommunicable disease risks:

First, cross-border externalities increase noncommunicable disease risk exposure. Examples include influences from abroad, such as fast food chains or trans-border health impacts from air pollution.

Second, globally shared needs, such as managing private-sector care delivery, providing pro-poor integrated care models, or scaling-up access to essential medicines, require shared solutions.

Finally, there has been insufficient action to protect regulation, trade and marketing restrictions, and taxes that reduce noncommunicable disease risks from opposing powerful actors. Powerful advocacy to champion the response, a critical factor in other successful responses, for instance in the HIV epidemic, has so far been inadequate against those forces.^9^

## Opportunities for global functions

Only a fifth of global health assistance is directed to global functions, much less than needed and with too little focus on the shared benefits of noncommunicable disease prevention and management.^10^ Global functions for noncommunicable diseases must leverage global health law and governance, resource shared needs, and have strong, inclusive leadership.

### Global health law and governance

Law and governance mechanisms are important, but underutilized global functions to address domestic and cross-border commercial noncommunicable disease determinants and externalities. The Framework Convention on Tobacco Control has been critical for mitigating the harmful effects of globalized tobacco, but unevenly implemented.^11^ The World Health Organization (WHO) *Global strategy to reduce the harmful use of alcohol*^12^ and the *WHO Action plan for the prevention and control of NCDs 2013–2020*^13^ recommendations for evidence-based, cost–effective interventions addressing unhealthy consumption of alcohol and calorie-rich foods, have also been applied unevenly.

Non-binding, so-called soft law instruments, such as global guidance and standards, influence societal norms, corporate conduct and legislative and policy priorities.^14^ There is a scope for the provision of more guidance on how to regulate damaging industries. Global functions should also address common legal barriers to noncommunicable disease prevention, for example reforming discriminatory laws counterproductive to achieving universal health coverage (UHC) or providing funding and capacity-building that enables countries to respond to trade law challenges.

Global governance and accountability mechanisms have been established to accelerate progress towards sustainable development goal (SDG) target 3.4, “by 2030, reduce by one third premature mortality from non-communicable diseases through prevention and treatment and promote mental health and well-being.” The SDGs explicitly include noncommunicable diseases, but their inclusive scope may limit attainment without strong leadership and funding. While legal and governance mechanisms are promising, inadequate funding and lack of political will continue to present barriers to addressing commercial noncommunicable disease determinants.

### Global public goods for shared needs

Determining health priorities and allocating resources at the national level is the work of sovereign states; however, the global community could do much more to address shared noncommunicable disease needs.

Multiparty agreements and platforms have effectively disseminated knowledge, lowered prices, increased access and evaluated the effectiveness of technologies for infectious, child and maternal diseases. Examples of such agreements are the Clinton Health Access Initiative,^15^ and the Global Health Technologies Coalition.^16^ These now familiar global public goods can be retooled for noncommunicable diseases, for which such initiatives are few, recent, under-resourced and still unproven, such as the Coalition to increase access to noncommunicable disease medicines and products initiative.^17^

Health promotion is critical in the long-term, but greater primary health-care and UHC investments are needed to manage the current noncommunicable disease burden. While there are several ways to achieve UHC, sharing experiences globally, such as that of the Joint learning network, can assist countries in improving and scaling up their UHC and health system strengthening efforts.^18^

The global research community provides valuable data that enlighten our understanding of noncommunicable disease prevention and control.^19^ However, even though major donors have supported global noncommunicable disease research, such as the Global Alliance for Chronic Diseases,^20^ research and development funding allocated to noncommunicable diseases in low- and middle-income countries is low and mostly focused on questions more immediately relevant to rich countries, such as cancer treatments with low cost–effectiveness.

### Championing a global response

The field of noncommunicable disease needs stronger, more visible global leadership. Both strong advocacy and leadership and increased funding can create a virtuous cycle that further leads to greater actor power and effectiveness. Notable and well-funded cases are The Global Fund to Fight AIDS, Tuberculosis and Malaria, and Gavi, the Vaccine Alliance.

Public health leaders within the noncommunicable disease field, such as the United Nations interagency task force on the prevention and control of noncommunicable diseases, or the WHO Global coordination mechanism on the prevention and control of noncommunicable diseases, have a narrow focus and are inadequately supported. As a result of these weaknesses, such mechanisms are not well equipped to manage the broad scope and partnerships needed for a successful global noncommunicable disease response. This situation has even prompted calls for the creation of a new independent public–private partnership to take the lead in the noncommunicable disease response.^21^

A strong, unified voice for people living with noncommunicable diseases that could leverage the enhanced governance mechanisms discussed above would also add pressure on powerful vested interests opposing disease control measures.

## Conclusion

The main response to noncommunicable diseases must take place downstream at the country level, but the overwhelming cost of country-specific functions has discouraged donor action. Global functions may represent comparatively smaller, upstream investments that donors can provide to all countries, while still allowing them autonomy to set their own priorities.

The tasks ahead are to further define the global functions required for noncommunicable diseases and to quantify their costs and impacts. Clean air, healthy food and living spaces, freedom from coercive messages, access to high quality care and protection for vulnerable populations, should be the focus of global functions. Since these are global phenomena, they are everybody’s business.
